# Characterization of an endogenous retrovirus class in elephants and their relatives

**DOI:** 10.1186/1471-2148-4-38

**Published:** 2004-10-11

**Authors:** Alex D Greenwood, Claudia C Englbrecht, Ross DE MacPhee

**Affiliations:** 1 GSF-National Research Center for Environment and Health, Institute of Molecular Virology, Ingolstädter Landstr. 1, D-85764 Neuherberg, Germany; 2 Department of Vertebrate Zoology, American Museum of Natural History, Central Park West at 79^th ^Street, New York, New York 10024-5192 USA; 3 GSF-National Research Centre for Environment and Health, Institute of Bioinformatics, Ingolstädter Landstr. 1, D-85764 Neuherberg, Germany

## Abstract

**Background:**

Endogenous retrovirus-like elements (ERV-Ls, primed with tRNA leucine) are a diverse group of reiterated sequences related to *foamy *viruses and widely distributed among mammals. As shown in previous investigations, in many primates and rodents this class of elements has remained transpositionally active, as reflected by increased copy number and high sequence diversity within and among taxa.

**Results:**

Here we examine whether proviral-like sequences may be suitable molecular probes for investigating the phylogeny of groups known to have high element diversity. As a test we characterized ERV-Ls occurring in a sample of extant members of superorder Uranotheria (Asian and African elephants, manatees, and hyraxes). The ERV-L complement in this group is even more diverse than previously suspected, and there is sequence evidence for active expansion, particularly in elephantids. Many of the elements characterized have protein coding potential suggestive of activity.

**Conclusions:**

In general, the evidence supports the hypothesis that the complement had a single origin within basal Uranotheria.

## Background

ERV-Ls are retroviral elements (retroelements) lacking the envelope gene (*env*) and exhibiting homology to the class of human endogenous retroviruses designated as HERV-L by [1]. Similar retroelements have been identified in several eutherian groups (see below), but their incidence in metatherians and monotremes is not known at present. They presumably arose from successful germ-line infection by *foamy*-like viruses, but when or how many times this might have occurred during the course of eutherian evolution is unknown. From the perspective of evolutionary biology it is of great interest that some classes of ERVs are known to retain original functions, including the capacity to produce infectious viral particles [2]. Others have gained novel regulatory functions in the mammalian genome [3]. Formation of the human placenta may depend on expression of a HERV-W element *env *gene [4]. Human immunodeficiency virus (HIV) shares specific functionally homologous sequences with endogenous retroviruses, suggesting the possibility that recombination with ERVs could change the properties of exogenous retroviruses [5]. Thus, ERVs may serve as a variable pool from which exogenous viruses may diversify. Exogenous retroviruses may have originated from ERVs and ERV-Ls in particular may represent an intermediate between retrotransposons and exogenous viruses [6].

Comparison of ERV-L polymerase (*pol*) gene sequences from 22 mammalian species revealed ERV-Ls that have expanded in copy number and remained active over long periods of time [1]. Phylogenetic analysis of these sequences demonstrated that primates and rodent ERV-L sequences are both diverse and, with few exceptions, monophyletic, whereas carnivore and ungulate ERV-L sequences were polyphyletic. The phylogenetic picture reflects the particularly robust expansion of the primate and rodent ERV-L complement. Importantly, the primates and rodents were the only groups that included ERV-L sequences with protein coding potential and therefore potential transpositional activity. These points suggests that, if the history of active expansion of retroelements within a group can be deciphered, it might be possible to use this information in the same way that parasite data are conventionally used [7], to perform tests of host phylogenetic relationships that are at least logically independent of other data sources. In this connection, the superorder Uranotheria is of particular interest.

Uranotheria [8] is the most recent nomen for a constellation of relationships that has, in fact, been supported by the majority of ungulate specialists throughout the past century. Simpson [9] grouped proboscideans, hyraxes, embrithopods and sirenians under the group-name Paenungulata, but was not certain of its monophyly. Most other authorities have supported this clade, albeit with some variation in content, in the years since Simpson's [9] publication (e.g., [10-14]). McKenna and Bell [8] divided Uranotheria into three major groups, Hyracoidea, Embrithopoda, and Tethytheria. The last is further subdivided into Sirenia and Behemota; behemotans consist of Proboscidea and Desmostylia. Only Hyracoidea, Sirenia, and Proboscidea possess living members.

Morphologically, there is considerable evidence that supports the association of Proboscidea and Sirenia as sister taxa to the exclusion of Hyracoidea [10], and little that appears to contradict it. Fischer and Tassy [15] take the position that alleged hyracoid morphological resemblances to tethytheres are either convergences or misconstrued, on the argument that hyraxes are in fact perissodactyls or closely allied to them. This aspect of the Fischer-Tassy hypothesis is not supported by molecular data [14,16]. On the other hand, it must also be admitted that sequence data have not provided especially strong support for Tethytheria (and, by extension, the monophyly of Uranotheria) [17]. In the most recent exercise in this arena, Asher et al [18] were able to recover Tethytheria under certain conditions when fossil and morphological data were combined with sequence information, but not when sequence data were used alone.

To investigate whether ERV-L and other retroelements may be useful in resolving phylogenetic questions involving uranotheres at multiple taxonomic levels, we utilized an ERV-L polymerase gene (*pol*) fragment using degenerate primers tested in other mammalian orders. Extending our previous work [19], we found that ERV-L sequence diversity was high in all members of this group and that phylogenetic analysis of our data to a limited extent supported Uranotheria as a distinct clade when sequences that lack coding potential are used. By contrast, sequences that are potentially active form separate monophyletic groups, indicating a more recent origin. Thus, it appears that ancient ERVs reflect the phylogeny of their host like classic genes and more recently active ERVs will tend to be more similar to one another as opposed to their host.

## Results

### Among-clone comparisons

A ~330 bp PCR product was amplified for African elephant, Asian elephant, manatee, and rock hyrax. The products were cloned and 10 clones sequenced for each product. Of the 40 sequences thus developed, only one Asian elephant sequence had no homology to ERV-Ls and was removed from analysis (not shown). No identical sequences were shared among taxonomic groups. All nine Asian elephant and all 10 manatee clones were unique. However, one Asian elephant clone, designated Max3 (accession number AY394573), was a recombination product of clone Max2 (accession number AY394572) and clone Max6 (accession number AY394576). Whether this represents a PCR artifact or is a genomic recombination event is not known. However, it is not expected that recombinational PCR would be observed in modern undamaged DNA [20].

Among the African elephant runs, four clones differed at 0–1 positions. As PCR errors probably account for these minor differences we assume only 6 unique ERV-Ls were discovered for this individual. Similarly, the hyrax sample yielded 3 groups comprised of 2 identical sequences, while two other sequences differed at 5 positions. Thus, 5 unique ERV-Ls were also obtained for *Procavia*.

**Figure 1:**
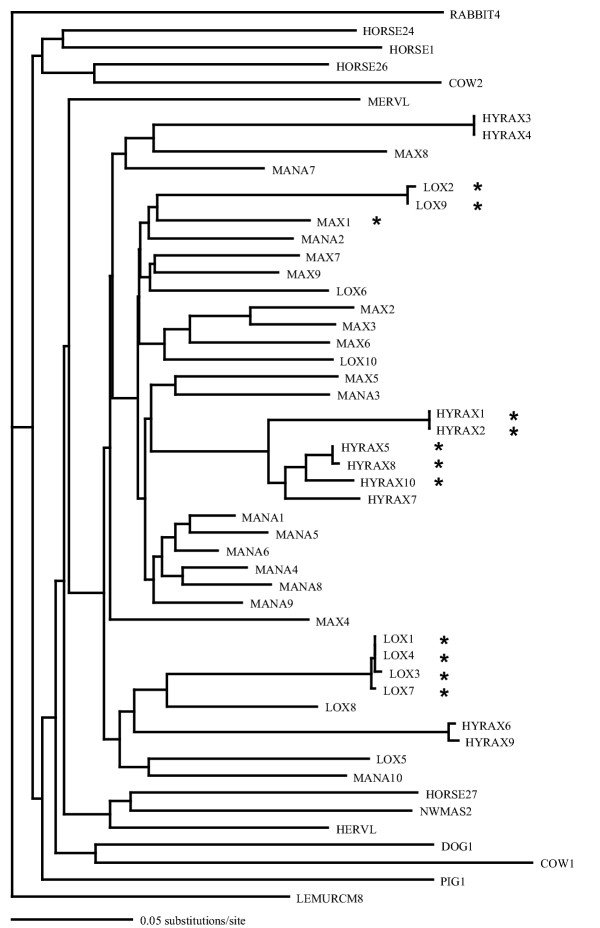
Endogenous retrovirus type L (ERV-L) phylogeny. Non-uranothere sequence designations taken from [1]. Uranothere designations are, Max (*Elephas maximus*), Lox (*Loxodonta africana*), Mana (*Manatus trichechus*), and Hyrax (*Procavia capensis*). Neighbor-joining tree of all uranothere sequences including representative ERV-L elements from other mammalian orders. "*" designates sequences with coding potential.

Recovered sequences were compared to a mouse element with full coding potential in the *gag *and *pol *genes (MuERV-L, GenBank no. Y12713). Twelve clones were in frame with no stop codons. However, only 6 of the total 12 were unique (Figure 1). This is surprising, as 87 sequences from 22 mammalian species previously revealed only 7 sequences with coding potential [1]. Among the 39 sequences determined here, 6 unique sequences had coding potential among only 4 species. The observed sequence diversity and frequency of observed coding potential is consistent with active ERV-L expansion in these four species and consistent with results with a smaller internal fragment from the same groups (plus extinct woolly mammoth in the proboscidean sample) [19].

### Phylogenetic analysis

**Figure 2:**
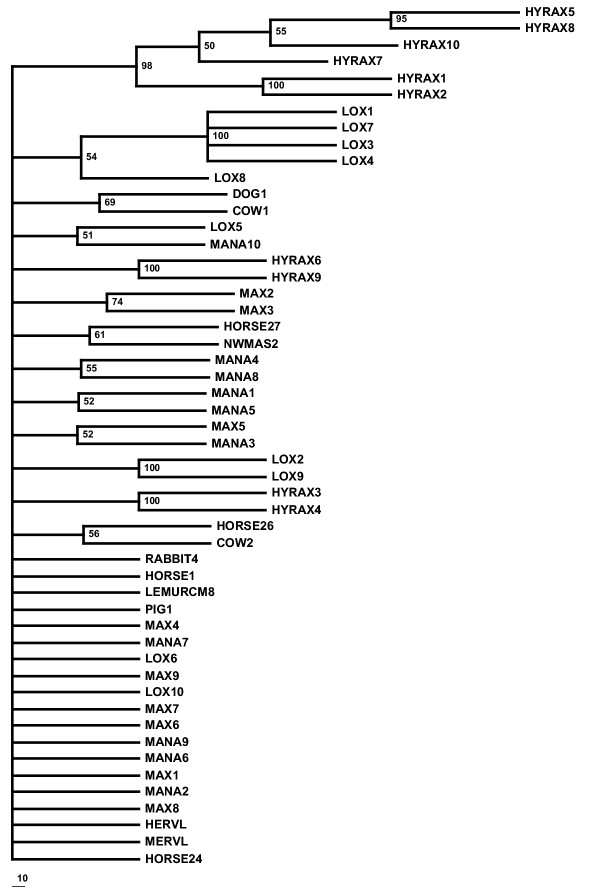
Maximum parsimony bootstrap consensus tree of all uranothere ERV-L sequences. Bootstrap values over 50% are shown. The scale bar indicates the number of steps.

**Figure 3:**
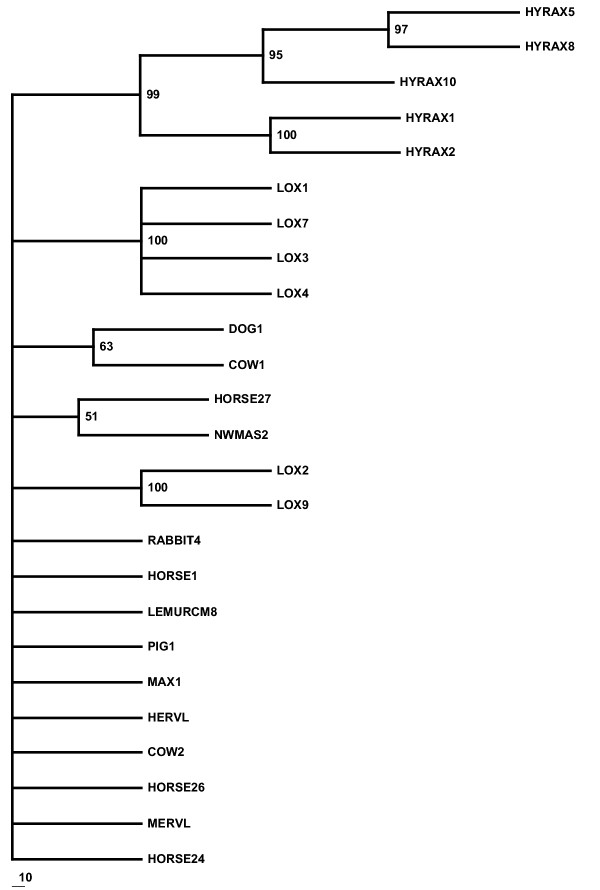
Bootstrap consensus tree of Uranothere ERV-L sequences with coding potential only.

**Figure 4:**
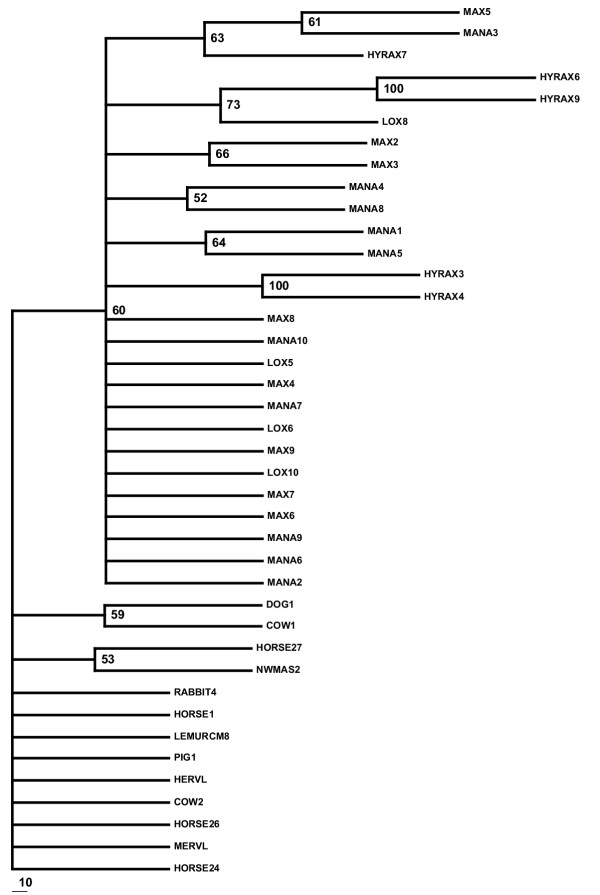
Bootstrap consensus tree of Uranothere ERV-L sequences without coding potential.

**Figure 5:**
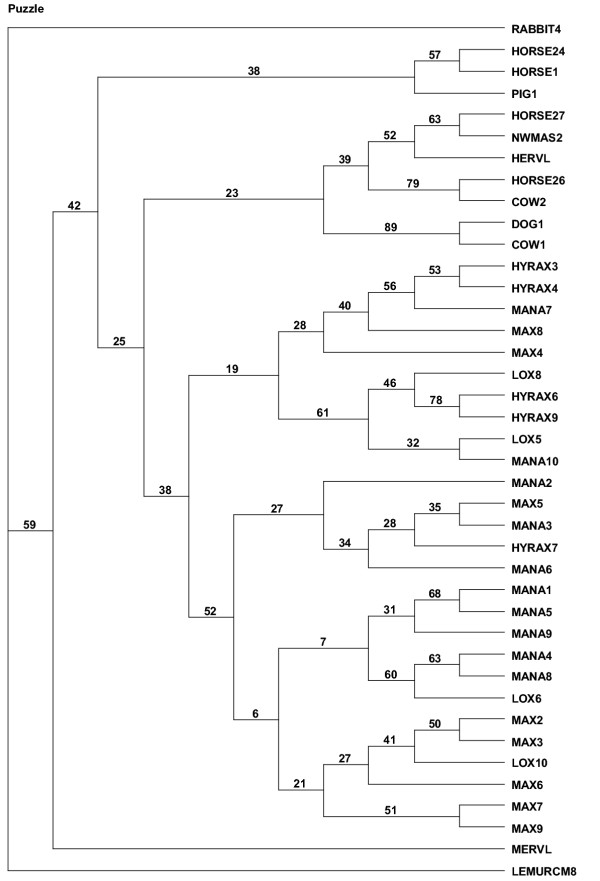
Quartet Puzzle maximum likelihood tree of sequences without coding potential. Puzzle support for each node is indicated.

A heuristic search of the entire uranothere ERV-L data set yielded 12 equally parsimonious trees, a strict consensus of which (Figure 2) showed poor recovery of accepted clades within Uranotheria and low bootstrap support at each node. While neighbor-joining analysis produces a tree with uranothere sequences as a monophyletic group, branch lengths in some cases were very short and bootstrap support under any method used was not statistically significant (Figure 1). On the assumption that sequences with potential RT activity may have been under different evolutionary constraints and may differ in their phylogenetic resolution, the sequences with no stop codons in the retrieved *pol *gene were analyzed separately from those with stop codons (Figure 3, 4 and 5). Those with potential RT activity grouped as distinct monophyletic groups, possibly reflecting their more recent activity and thus showing closer affinity to one another as opposed to other related ERV-L sequences (Figure 3). Those with stop codons showed a different picture with modest support for Uranotheria as one might expect for single or low copy sequences that have been transmitted vertically over time (Figure 4). Likelihood analysis of the data produced similar groupings, though with weak support, suggesting the associations found are not an artifact of the phylogenetic analysis methodology (Figure 5). However, ME analysis did not produce statistically significant resolution for any ERV group examined including non-uranothere ERVs (not shown). Poor resolution within Uranotheria with all analyses could be due to several factors, although the likeliest is different ages of individual element copies. This is not unlike the situation with various sequences recovered from primates and rodents: some sequences reconstruct accepted ordinal groupings, while others do not. For example, in the study by Bénit et al. [1], one New World monkey sequence (As2) grouped with two dog sequences and not with other primates. In other mammals, sequences (e.g., those retrieved from cow and horse) were dispersed in no evident pattern.

## Discussion

The most important finding resulting from this study is that elements that have undergone expansion–i.e. have remained transpositionally active–are the ones that are most likely to group monophyletically and those that have not tend to be consistent in their higher-level taxonomic distribution (in this case, at the superordinal level). Thus, there are different elements within the same family demonstrating different evolutionary trajectories. It is reasonable to suppose that for some groups, such as rodents, primates, and uranotheres, continued expansion of active ERV-L elements was tolerated by the host. By contrast, in other ungulate and carnivore lineages active elements were not inherited or were silenced early during their evolution and ERV-L expansion did not occur. Older elements tend to evolve as typical orthologous sequences. An advantage of the great diversity of elements is that with a single PCR, cloning, and determination of multiple clone sequences one retrieves multiple independent sequences with which to do phylogenetic analysis. In these regards, the uranothere evidence is consistent with results previously reported for primates and rodents in which older elements and elements that have undergone bursts of transposition were found coexisting. In each of these groups there is now good sequence evidence for retroelements that have retained coding capacity, which is of some interest since HERV-L *pol *(for example) is known to be expressed in specific tissue types [21]. This indicates that, in addition to potential transpositional activity, retroelements other than syncytin may have acquired biological functions important for their hosts.

## Conclusions

In terms of our general results, elephants and the manatee were found to contain the most diverse sequences, while hyrax showed comparatively less diversity. This finding is consistent with results from our previous study involving extinct elephantids [19]. Elements that have been investigated in other ungulates (bovids and suids) do not yield monophyletic groupings and are represented by low copy numbers [1], suggesting that expansion did not occur (or has not recently occurred) in these taxa. Although critical studies would have to be undertaken to demonstrate the matter conclusively, it appears that amplification and diversification of ERV-L elements were independent events in primates, rodents and uranotheres.

Although this study shows that the value of ERV-Ls for the narrow purpose of phylogenetic reconstruction is limited at higher taxonomic levels, ERV-L is only one class among many different groups of ERVs in mammalian genomes. Some regions of ERVs have been used successfully to reconstruct phylogenies at lower taxonomic levels [22]. Additional transposable elements could serve as phylogenetic markers in a manner similar to ERV-L in the present study, while providing multiple independent sequences to test ordinal level phylogenies.

## Methods

### Samples

African elephant (*Loxodonta africana*) DNA was supplied by N. Georgiadis of the M'Pala Research Centre, Kenya. Asian elephant (*Elephas maximus*) blood was provided by J. Hektor of the Tierpark Hellabrunn, Munich. Manatee (*Trichechus manatus*) blood was provided by D. Murphy of the Lowry State Park, Florida. Hyrax (*Procavia capensis*) muscle was provided by G. Amato of the Bronx Zoo, New York.

### DNA extractions

One ml of blood or approximately one gram of tissue was incubated in 1–2 ml 10 mM Tris-/Cl (pH 7.5), 10 mM EDTA (pH 8.0), 50 mM NaCl, 2% SDS, and 0.6 mg/ml Proteinase K overnight at 37 C, extracted with phenol and chloroform, and subsequently concentrated with 50 ul Millipore Ultrafee MC 30,000 NMWL columns or precipitated in 2.5 volumes ethanol and 1% NH4 Oac.

### PCR, cloning, and sequencing

PCR primers for the ERV-L *pol *gene are described in [1]. Three μl of extract was added to 50 μl PCR containing standard buffer supplied by Boehringer Mannheim and 30 PCR cycles performed. PCR products were cloned using the pGEM-T cloning system (Promega). After heat shock into bacteria, ampicillin and blue/white selection, colonies were picked with a sterile pipette tip and added to 30 μl PCR reactions where M13 forward and reverse primers were used to amplify inserts for 25 cycles using the same buffer system described for ERV-L amplifications and as described in [19]. Five μl of the colony PCR products were visualized on ethidium-stained gels. Insert positive PCR reactions were purified with QIAquick columns and sequenced with T7 and SP6 primers using an ABI 377 sequencer.

### Phylogenetic analysis

#### Alignment

Representatives of each clade determined by Bénit et al. [1] were included in an alignment with the elephant, manatee, and hyrax sequences determined. HERV-L, X89211; MERV-L, Y12713; NWM (AS2), AJ233633; Lemur CM8, AJ233645; horse1, AJ233650; horse24, AJ233654; horse26, AJ233655; horse27, AJ233656; pig1, AJ233661; cow1, AJ233662; cow2, AJ233663; dog1, AJ233665; rabbit4, AJ233627. Alignments were performed using ClustalX [23] and adjusted where necessary. Elephant, manatee and hyrax sequences have been deposited in GenBank (accession numbers AY394571-AY394609)

#### Phylogenetic methods

Maximum parsimony and neighbor joining analysis was performed using PAUP 4.0b [24]. Heuristic searching including all uranothere ERV-L sequences yielded 12 maximum parsimony trees (MPTs). 100 bootstrap replicates were performed to test MPT robustness, the strict consensus of which is shown in Figure 2. A bootstrap consensus tree using only sequences with no stop codons yielded 2 trees. The strict consensus of these trees following 1000 bootstrap replicates is shown in Figure 3. A consensus tree of 1000 bootstrap replicates, this time excluding sequences with coding potential, is shown in Figure 4. Maximum likelihood was performed using quartet puzzle in PAUP 4.0b after determining the evolution model as HKY +G using Modeltest 3.5 [25]. 10,000 puzzling steps were employed to determine the tree topology. Minimum evolution trees were generated using the program Mega2 [26].

## Authors' contributions

ADG oversaw the molecular genetic studies, coordinated the study, participated in the sequence alignment and phylogenetic studies and co-drafted the manuscript. CCE participated in the phylogenetic analysis. RDEM participated in the design and coordination of the study and co-drafted the manuscript.
